# Macronutrient Adequacy of a Mediterranean-type Meal Examined at Recommended and Below Recommended Energy Values

**DOI:** 10.26502/jfsnr.2642-110000129

**Published:** 2023-05-10

**Authors:** Alan M. Preston, Priscilla K. Clayton

**Affiliations:** 1 Department of Biochemistry, University of Puerto Rico, Medical Sciences, Campus, San Juan, PR 00936, USA; 2 Department of Nutrition and Dietetics, Florida, International University, Miami, FL 33199, USA

## Abstract

**Background:**

Mediterranean style eating pattern is regarded as among the worlds’ healthiest. Numerous studies have shown that the Mediterranean eating pattern can promote weight loss, however, if combined with caloric restriction as promoted via internet sites, are inherent advantages retained or do macronutrients fall below recommended levels and if so, at which energy values does this occur?

**Objective:**

To address this question

**Methods:**

We have formulated a meal which was developed from items on menus in Barcelona, Spain. Macronutrients were determined using NDSR software and the meal was assessed for carbohydrate, fat and protein content at recommended levels of 2500 and 2000 kcal/day as well as at 1600, 1200 and 800 kcal/day through control of portion sizes. Authenticity of the meal as being Mediterranean- type was verified by comparison to established standards contained in dietary guidelines for Americans as well as similarity to percent of macronutrients published in the literature.

**Results:**

Comparison of our results to guidelines for a Mediterranean style eating pattern showed fruit, protein and oil intake to be sufficient but not so for the vegetables, grains and dairy food groups. All macronutrients reached dietary recommended amounts when analyzed at energy values of 2500 and 2000 kcal/day. Fat and carbohydrate content satisfied recommended amounts at intakes of 1600 and 1200 kcal/day but the amount of protein was insufficient at all values below 2000 kcal/day.

**Conclusion:**

Although a Mediterranean-style eating pattern is among the healthiest, in order to maintain macronutrient adequacy, it should not be energy compromised.

## Introduction

Healthful eating promotes individual well- being as well as sound nutrition. High on the list of eating paradigms can be found in the southern part of Europe which encourages the consumption of fresh, seasonal, and local foods. The principal constituents of this diet include proportionally high consumption of olive oil, legumes, unrefined cereals, fruits, and vegetables, moderate to high consumption of fish, moderate consumption of dairy products (mostly as cheese and yogurt), moderate wine consumption, and low consumption of non-fish meat. This pattern of eating has come to be called “Mediterranean” [1,2] which is, associated with nutritional adequacy [3], and weight loss plus cardiovascular risk factor level reduction as comparator diets in overweight or obese individuals [4]. Acceleration of weight loss, as suggested in internet sites can be accomplished by coupling a Mediterranean diet with energy reduction to 1500 and 1200 kcal/day [5] which raises the question - at what reduction level, if any, is macronutrient adequacy of the Mediterranean diet jeopardized. To answer this, we have examined a typical Mediterranean meal for macronutrient content using nutritional analysis software at daily energy values of 2500, 2000, 1600, 1200 and 800 kcal. The aim is to identify the most vulnerable macronutrients and the energy values at which deficiency is first noted.

## Methods

### Diet Selection

To evaluate the Mediterranean diet, it should be mentioned that depending upon the country, subtle differences in composition exist [6]. The Greek variant has high monounsaturated/saturated fat ratio, moderate alcohol consumption, high consumption of legumes, grains, cereals, fruits and vegetables, low consumption of meats and modest dairy consumption. The Italian variant of the Mediterranean diet is characterized by higher consumption of pasta and slightly more wine whereas in Spain fish consumption is particularly high. It should be mentioned that this eating pattern can also be found in Portugal and South France [1] but not to an equivalent extent. We have selected the Spanish variant for our analysis, however ingredients are not all encompassing since the regions and seasonal availability of foods in each district has its own particularities. In our case, we collected menus in the winter of 2021 from a variety of restaurants and tapas shops in greater Barcelona specializing in “Mediterranean” cuisine. Our formulations are therefore representative of the Catalonia area but not necessarily the entire Spanish nation and are presented in table 1.

Wines are: Red wine (Cab Sauvignon) lunch, White wine Pinot) dinner.

Popular weight reduction plans are often structured as to caloric limitation with 1600 (reduced calories), 1200 (low calories) and 800 (very low calories) being among the most frequent types [7]. Therefore, we have chosen these values for testing and compared them to the Dietary Guidelines for Americans (DGA’s)-suggested level of 2500 kcal/day [8] and the Dietary Reference Intake (DRI)-recommended level of 2000 kcal/day [9]. Table 2 is a summary of caloric distribution per meal and meal contents from Barcelona eateries (shown in Table 1) containing 2500 kcal. Table 3 is a summary of caloric distribution using the same meal containing 2000, 1600, 1200 and 800 kcals which were obtained through adjustment of portion sizes.

### Diet Analysis

The dietary analysis was caried out using the Nutrition Data System for Research (NDSR), 2019 version [10]. Macronutrient content which includes energy containing components are expressed as DRI values which give an approximation of how much fat, carbohydrate among others should be eaten, based on an average- sized adult doing an average amount of physical activity [9].

## Results

An important consideration is that meals in our study were selected from eateries, which although similar to meals served at home, may not be characterized as truly Mediterranean. Numerous surveys, conducted in the United States and Europe report unhealthier eating patterns away from home with higher caloric intake and poorer diet quality [11,12]. Whether these findings would apply to establishments specializing in Mediterranean cuisine deserves consideration. To address this issue, we have compared our eating pattern to “The Healthy Mediterranean- Style Pattern” as suggested in the latest edition of the DGA’s [8]. Table 4 shows recommended daily amounts of food groups (top line) and our results (bottom line).

Food Group- Recommended Amounts= top line Our Amounts= bottom line.

Our values exceed recommended amounts for fruits, protein (notably, mostly fish and no red meat) and oils. Our values fall short for the vegetables, grains and dairy groups.

The percentages in table 5 fall within published ranges for Mediterranean-based meals, which are: Carbohydrate = 40-65%, Fat = 28-40% and Protein = 10-35% [13]. For alcohol, suggested amount is two 5 oz glasses of wine/day for men and one 5 oz glass of wine/day for women [8]. Results in table 6 show that saturated fat and added sugars satisfy DRI recommendations at all energy values. The amount of fat exceeds recommendations at the 2500 kcal level and falls below recommendations at the 800 kcal level. Carbohydrates are adequate at all levels except 800 kcal intake. Protein insufficiency begins at levels below 2000 kcal.

## Discussion

When selecting our meals and eating times we have relied on the traditional patterns of greater Barcelona akin to the early-mid twentieth century. “Mediterranean” encompassed more than just diet but a particular lifestyle established over many years [6,13]. Meals had been communal and were seen as important opportunities to talk and be with family and friends. A “siesta” often followed the main meal, at lunch when commercial activity was suspended. Unfortunately, current lifestyles are becoming more “westernized” accompanied by increased cardiovascular problems and obesity [14–16]. A recent nutrition survey in Spain reported compliance at only 40% [17].

In the present study, results in table 4 do not always satisfy ideal recommendations. It must be remembered that our results represent only one day’s intake. Since a Mediterranean eating pattern is comprised from a robust selection of recipes [18] over a period of time, it is reasonable to assume that other days in the week, with different menus, will have greater amounts of vegetables, dairy and grains as well as lesser amounts of fruits, protein and oils. So, the excesses and deficits should balance out and in the long term, amounts should satisfy the recommended Mediterranean pattern.

Results from table 5 further affirm compliance with a Mediterranean-type meal for all energy values. On the positive side, saturated fatty acids are below the limit of 8% [13] and monounsaturated fatty acids, due to abundant olive oil, comprise the overwhelming percent of total fatty acids. The small amount of trans fatty acids come from the codfish fritters and anchovies. Likewise, percent added sugars (all from flan) fall below the suggested limit of 20% [13]. Finally, there is a reasonable balance of total protein between the animal and vegetable forms. Results from table 6 answer our question of which macronutrients fall below DRI recommendations and the energy value at which this occurs. In order of energy level: protein inadequacy began at values below 2000 kcal, and carbohydrate and fat were insufficient at values less than 1200 kcal. It would be of interest to perform this study with micronutrients, however, validation of normal intake of lesser-consumed nutrients would require several weeks of menu analysis [19].

Strengths of this study include use of actual menus from Spanish restaurants and not suggested recipes from websites, realistic portion sizes and data analysis using NDSR software. Weaknesses are that one day’s meal composition cannot satisfy all criteria of a Mediterranean eating pattern and whether one day’s data collection is sufficient to represent typical macronutrient content is subject to question. Foster et al. [20] estimated that 2 days of 24 hr dietary recalls are sufficient for assessment of macronutrients so while valid for this particular meal, the same conclusions for other meals of this type might not be justified. Finally, it should also be noted that this is a representative meal and meals of different composition could alter results but not likely enough to change macronutrient adequacy.

## Conclusions

We have formulated a daily meal typically consumed in greater Barcelona, Spain and assessed it for fidelity to a Mediterranean eating pattern. By adjusting portion size, we evaluated the compliance of macronutrients to recommended DRI’s at energy levels of 2500, 2000, 1600, 1200 and 800 kcal. Although the Mediterranean eating pattern is among the most healthful, we found that in our case, energy intake less than 2000 kcal results in the amount of protein to fall below the suggested DRI. In addition, below 1200 kcal, the DRI’s for fat and carbohydrate will not be satisfied. Consequently, using a Mediterranean-type meal, as presented here, should not be calorically manipulated as a method of weight reduction.

## Figures and Tables

**Table 1:** Typical Catalonian Eating Plan

Time	Meal	Size	Meal Selection
8-9am	Breakfast	Small-Medium	Coffee with milk
10-11am	Snack	Small	Garlic olives
2pm	Lunch	Small Portions	Goat cheese salad
			Codfish fritters
			Provincial braised hake
			Spanish fruit salad
5pm	Snack	Small	Anchovies in vinegar
9-10pm	Dinner	Moderate (Small Portions)	Catalan salad
			Sauteed chicken breast
			Spanish Flan

Daily beverages- 1.8liters water (8x8 oz glasses = 64oz) 250-300ml wine

**Table 2:** Caloric Distribution per Meal + Meal Content for 2500 kcal^*^

Breakfast	Morning Snack	Lunch	Afternoon Snack	Dinner
375kcal	125kcal	1,000kcal	125kcal	625kcal
2 items^a^	1 item^b^	4 items^c^	1 items^d^	3 items^e^

* Beverage (wine+water) = 250kcal (125kcal each- lunch and dinner)

a Coffee+Accompanient

b Fruit, nuts, cheese, tapas

c One main course, two side course, one desert

d One main course, one side course, one desert

All courses contain realistic course portion sizes

**Table 3:** Caloric Distribution per Meal Content for 2000^1^, 1600^2^, 1200^3^ and 800 kcal^4^

Breakfast	Morning Snack	Lunch	Afternoon Snack	Dinner
300 kcal	100 kcal	800 kcal	100 kcal	500 kcal
240 kcal	80 kcal	640 kcal	80 kcal	400 kcal
180 kcal	60 kcal	480 kcal	60 kcal	300 kcal
120 kcal	320 kcal	320 kcal	40 kcal	200 kcal

1 First Row

2 Second Row

3 Third Row

4 Fourth Row

**Wine :** 200 kcal, 160 kcal, 120 kcal and 80 kcal

Water remains at 1.8 liters/day

Number of items are the same, but portion sizes are smaller

**Table 4:** Mediterranean-Style Dietary Pattern Fed at 2500 kcal

Vegetables	Fruits	Grains	Dairy	Protein	Oils
3.25 cup eq	2.5 cup eq	8.5 oz eq	2.5 cup eq	7.5 oz eq	3.3g
2.8 cup eq	2.9 cup eq	7.8 oz eq	0.91 cup eq	8.8 oz eq	143g

**Table 5:** Caloric Distribution-macronutrients

% Fat =	35		% CHO =	41.9		added sugar	17.80%
% SatFA =	6.6	18.9%*	% Protein =	13.2			
% MUFA =	22.8	65.10%	% Animal =	7.9	60%**		
% PUFA =	5	14.30%	% Vegetable =	5.3	40%		
% Trans FA =	0.6	1.70%	% Alocohol	9.9			

* % of total FA’s

** % of total protein

**Table 6:** Amounts of Macronutrients at Energy Values from 2500 to 800 kcal

Nutrient	DRI	2,500	2,000	1,600	1,200	800
Fat g	44-77	99.8	79.9	63.9	47.9	31.9
Sat FA’s g*	< 30	18.3	14.6	11.7	8.8	5.9
Carbohydrate	130	271.7	271.4	173.9	130.4	87
Added sugars g	50	25.5	20.4	16.3	12.2	8.1
Protein g	56	83.7	66.9	53.6	40.2	26.8

* American Heart Association recommends 5-6% of total fat or <13 g [11]
